# Non-pharmacological interventions for opioid use disorder: protocol for a systematic review and network meta-analysis

**DOI:** 10.3389/fpubh.2026.1901002

**Published:** 2026-09-15

**Authors:** Miaomiao Li, Deyu Miao, Qingcong Mo, Qinghua Chen, Liming Lu

**Affiliations:** 1 Medical College of Acu-Moxi and Rehabilitation, Guangzhou University of Chinese Medicine, Guangzhou, Guangdong, China; 2 South China Research Center for Acupuncture and Moxibustion, Guangzhou University of Chinese Medicine, Guangzhou, Guangdong, China; 3 The First School of Clinical Medicine, Guangzhou University of Chinese Medicine, Guangzhou, Guangdong, China

**Keywords:** network meta-analysis, non-pharmacological intervention, opioid use disorder, withdrawal, randomised controlled trial, protocol

## Abstract

**Introduction:**

Non-pharmacological interventions (NPIs) are increasingly recognized as essential adjuncts to alleviate symptoms and enhance compliance in opioid use disorder (OUD). Although clinical guidelines recommend specific NPI modalities, high-quality evidence regarding their comparative hierarchies and time-course dynamics remains limited. We plan to conduct a systematic review and network meta-analysis (NMA) to evaluate and rank the efficacy of diverse NPIs.

**Methods and analysis:**

PubMed, Embase, CENTRAL, and CINAHL will be searched without language restrictions. We will include randomized controlled trials (RCTs) evaluating adult OUD patients randomized to commonly utilized NPIs versus standard pharmacological care (e.g., methadone, buprenorphine) or active alternative NPIs. Reviewer pairs will independently screen studies, extract data, and assess the risk of bias using the Cochrane RoB 2.0 tool, implementing a strict decision rule where failures in allocation concealment or missing data domains automatically trigger an overall high risk-of-bias judgment. Frequentist multivariate random-effects NMA (REML method) will be conducted to synthesize outcomes across primary (abstinence, craving, treatment retention) and secondary (relapse rates, anxiety, depression, sleep quality) endpoints. Node-splitting models and network meta-regressions will explore critical effect modifiers, focusing specifically on background pharmacological regimens and time-course treatment effects across distinct intervention timepoints. Finally, the GRADE framework will be implemented via a minimally contextualized approach to structure NPIs into hierarchical clinical tiers based on effect estimates and evidence certainty.

**Justification:**

This NMA will establish an evidence hierarchy for non-pharmacological OUD management by evaluating clinical efficacy and systematically stratifying optimal interventions based on their certainty of evidence (moderate-to-high versus low-to-very-low), thereby providing a standardized foundation for individualized clinical care and future trials.

**Trial registration:**

https://www.crd.york.ac.uk/PROSPERO/recorddashboard, PROSPERO CRD420261330214.

## Introduction

Opioid Use Disorder (OUD) is a serious global health crisis responsible for profound morbidity and mortality (1, 2). Approximately 60 million people engaged in illegal opioid use globally, making OUD the leading cause of drug-related mortality by accounting for about 450,000 (75%) of the 600,000 annual drug-related deaths worldwide. The geographical impact of this crisis is devastating; for instance, opioids were linked to over 80% of drug-related deaths in Europe in 2017, and two-thirds of opioid users in Asia exhibit problematic use. Similarly, in the U. S., over 500,000 overdose deaths over the past 15 years have measurably reduced national life expectancy, with annual deaths surpassing 100,000 during the pandemic and projections suggesting that opioid-induced mortality could reach 165,000 by 2025. Beyond fatal overdoses, OUD is a chronic brain disease that fundamentally alters neural circuitry, frequently resulting in severe depression, dysphoria, and anhedonia (3, 4). Consequently, this creates a massive societal and healthcare burden that is further complicated by severe comorbidities, including blood-borne infections, pregnancy complications, and synthetic drug poisonings.

While Medications for Opioid Use Disorder (MOUD) remain a cornerstone in managing physical dependence and reducing mortality, their long-term clinical success is often hindered by high patient dropout rates and an inability to address the complex behavioral and psychological drivers of addiction (5). To bridge these critical gaps, current research strongly supports the integration of adjunctive non-pharmacological interventions(NPIs). Psychosocial treatments, particularly Contingency Management (CM) and Cognitive Behavioral Therapy (CBT), have demonstrated significant efficacy in reinforcing abstinence, improving medication adherence, and increasing overall treatment retention when combined with standard pharmacotherapy (6). Furthermore, emerging neuromodulation technologies, such as Transcranial Direct Current Stimulation (tDCS) and repetitive Transcranial Magnetic Stimulation (rTMS), offer promising, non-invasive methods to directly modulate the brain’s reward circuitry, thereby attenuating cue-induced opioid cravings and alleviating withdrawal symptoms (7). Currently, smartphone apps, telehealth, and text-messaging platforms are increasingly utilized to deliver psychosocial interventions. They overcome geographic and logistical barriers, increase privacy, and promote therapeutic alliance, with some interventions showing significant increases in days of opioid abstinence and treatment retention (8). Ultimately, a comprehensive, multi-disciplinary care model that synergizes pharmacological stabilization with targeted behavioral and neuromodulatory therapies is essential for achieving sustained remission and holistic recovery in patients with OUD (6).

While decades of research generally support combining non-pharmacological therapies with MOUD to improve clinical outcomes, critical reviews reveal profound methodological gaps in network systematic review is that hinder definitive conclusions (9, 10). This difficulty primarily stems from massive heterogeneity in study designs, intervention types, and paired pharmacotherapies. Furthermore, previous network meta-analyses (11, 12) have predominantly focused on drug use and treatment retention, with limited attention to broader, patient-centred rehabilitative outcomes such as psychological wellbeing (e.g., anxiety and depression) or harm reduction. In addition, while reviews of neuromodulation show early promise, the current literature is limited by an absence of studies exploring the efficacy of neuromodulation specifically during the long-term maintenance phase of OUD treatment (13).

To resolve these critical evidence gaps, this study will execute a rigorous frequentist network meta-analysis (NMA) structured around multivariate regression and node-splitting frameworks. Rather than generating a simple linear ranking, this study aims to systematically decode how the comparative efficacy of diverse NPIs is modulated across four domains: background opioid agonist treatments(OAT) modalities, methodological risk of bias, time-course effects during the treatment period, and baseline psychiatric comorbidity profiles, thereby delivering a granular, evidence-based decision matrix to directly inform future clinical practice guidelines for OUD.

## Methods and analysis

### Standardised reporting and registration

This systematic review and network meta-analysis protocols have been registered in PROSPERO (registration number: CRD420261330214) and reported in accordance with the Systematic Review and Meta-Analysis Protocols (PRISMA-P) 2015 guideline (14).

### Search strategy

A preliminary (pilot) search was conducted on 15 April 2026 in PubMed, Embase, CINAHL, and CENTRAL, without language restrictions, to refine and optimise the search strategy. The pilot results were used exclusively for strategy calibration and were not used for study selection. Based on these results, the final search strategy was established (Supplementary Table S1). The formal comprehensive literature search will be performed according to this finalised strategy, and the results will be reported in the final manuscript. We will also screen the reference lists of eligible studies and relevant reviews to identify additional randomized controlled trials (RCTs). For studies published in languages other than English or Chinese, full-text translation will be performed using professional translation services or native-speaking collaborators to ensure accurate data extraction.

### Eligibility criteria

The detailed framework governing our inclusion and exclusion criteria is presented in Table 1. We will include RCTs evaluating NPIs, either compared against alternative NPI nodes or head-to-head against medication-based control, for the clinical management of individuals diagnosed with OUD. Medication-based control is specifically defined as standard opioid maintenance treatment (e.g., methadone or other approved pharmacotherapies for OUD). This reference node comprises regimens where maintenance medication is administered either as a standalone therapy, paired with non-active/placebo procedures, or evaluated alongside another active pharmacological protocol to serve as the baseline comparator for NPIs. Diagnostic eligibility will be strictly benchmarked against formalized criteria, including any version of the Diagnostic and Statistical Manual of Mental Disorders (DSM) or the International Classification of Diseases (ICD). For earlier or pragmatic trials where specific diagnostic manuals are not explicitly articulated, eligibility will be established based on consensus with clinical addiction specialists, verifying that participants exhibited verified physical dependency, are actively undergoing opioid detoxification, or are enrolled in pharmacological maintenance programs (e.g., methadone or buprenorphine care).

**Table 1:** Eligibility criteria.

Domain	Criteria
Publication type	Original research reports
	Trial registrations for which no linked publication could be identified were eligible only if they provided outcome data.
	Conference abstracts, narrative reviews, comments, letters, or editorials were not eligible.
Study design	RCTs
	Quasi-experimental trials, adaptive design, cross-over design, or secondary analysis of RCTs were not eligible.
Population	Age≥18 years (on average or >50% of participants)
	Diagnosis of OUD, or opioid dependence or abuse, based on one of: Recognized diagnostic criteria (e.g., any version of DSM or ICD), Individuals were recruited from a detoxification clinic, from emergency care visits for any reason, Individuals who, at baseline, had both used opioids in the past month and experienced an opioid-related problem
	Studies will be excluded if they: enrolled patients who utilized opioids exclusively for medical purposes, including chronic non-cancer pain, cancer-related pain, or postoperative pain; (2) included participants with severe, untreated psychiatric disorders; or (3) involved patients with co-occurring severe alcohol, benzodiazepine, or stimulant use disorders (dependence).
Interventions	Any NPIs for the treatment of OUD. Eligible NPIs must feature clearly defined, manual-guided protocols. Individual or group-based.
Comparators	NPIs or Medication-based. Medication-based control is specifically defined as standard OUD opioid maintenance treatment (e.g., methadone). Administered either alone, with placebos, or combined with another pharmacotherapy to serve as the baseline NPI comparator. Comparisons between different formats of the same intervention were not eligible.
Outcomes	Primary outcomes Point abstinence rate Craving Retention Secondary outcomes Return to Drug Use Anxiety Depressive Quality of sleep (measured by the PSQI) Adverse events
Timepoints	Pre-intervention (i.e., baseline) Post-intervention (8 weeks) Follow-up (24 weeks) Network meta-analysis was performed separately at each time point. If two or more follow-up assessments were available within the same time interval, the assessment closest to the lower bound of the respective category was selected.
Setting	Inpatient, outpatient, or community-based treatment setting
Geography	No limitations
Publication language	No limitations

RCT, randomized controlled trial; OUD, opioid use disorder; NPI, non-pharmacological therapy; DSM, Diagnostic and Statistical Manual of Mental Disorders; ICD, International Classification of Diseases; PSQI, Pittsburgh Sleep Quality Index.

Trials ineligible for quantitative pooling will be retained for structured narrative synthesis rather than omitted. This independent subset included: (1) RCTs lacking standardized numerical outcomes for network estimation, and (2) the limited literature on the protracted opioid withdrawal phase was too sparse to form a stable network node. Their core findings on treatment compliance, therapeutic efficacy, and withdrawal mechanisms will be fully integrated into the discussion.

We defined the outcomes as intervention efficacy at the end of treatment. The primary efficacy outcomes will be: (1) point-prevalence abstinence, defined as a negative urinalysis at end-of-treatment or a specified follow-up time point. For missing data, we will apply the “missing = positive” principle, with sensitivity analyses to test robustness. (2) craving, assessed by various self-reported questionnaires; and (3) treatment retention. Treatment retention will be operationalized as the proportion (percentage) of randomized participants remaining in the trial. This outcome will be dynamically tracked across two timepoints: (1) post-treatment retention (the percentage of individuals completing the treatment phase) and (2) follow-up retention (the percentage of individuals retained at the 24-week post-treatment follow-up).

As secondary outcomes, therapeutic efficacy and safety will be evaluated. Relapse rate will be operationalized as the number of participants returning to drug use during the post-treatment follow-up period. Secondary psychological and physiological outcomes, including anxiety, depression, and sleep quality (measured via the PSQI), will be evaluated based on continuous scale scores at the end of treatment. Safety will be quantified as the number of participants experiencing at least one adverse event by the end of treatment.

We excluded studies that enrolled patients who used opioids for chronic non-cancer pain, cancer-related pain, and postoperative pain, which may show systematically different responses to interventional procedures. Furthermore, we excluded quasi-experimental trials, adaptive design, cross-over design, and secondary analysis of RCTs, as these study types may introduce design-related heterogeneity and compromise the comparability, internal validity, and consistency assumptions required for network meta-analysis. Conference abstracts will be screened but excluded from synthesis due to insufficient data for bias assessment and outcome extraction. To address potential publication bias, relevant abstracts will be documented narratively, and their impact will be explored in sensitivity analyses assuming non-significant results for missing studies.

### Study selection

All identified citations will be exported to and managed using NoteExpress(4.0.09855). Following the eligibility criteria, two trained reviewers will independently and in duplicate screen the titles and abstracts of retrieved citations, and subsequently review the full texts of studies considered potentially eligible. Disagreements will be resolved through consensus or, if necessary, via consultation with clinical experts who are blinded to the trial results.

### Data extraction

Two trained investigators will independently and in duplicate extract data using a pre-tested, standardized information form tailored to addiction medicine, with disagreements resolved through consensus. For all included studies, we will abstract OUD-specific study and participant characteristics (e.g., baseline substance dependence severity and detoxification status), detailed protocols of manual-guided NPIs, and configurations of medication-based controls (including methadone or buprenorphine dosages).

Regarding secondary continuous endpoints (anxiety, depression, and sleep quality), we will prioritize adjusted least-squares means or end-of-follow-up mean scores that account for baseline values to maximize statistical power. If such data are unavailable, change-from-baseline scores will be utilized, and when neither is reported, end-of-study scores will be extracted. For multi-arm trials involving multiple NPI arms and a shared medication-based control group, the shared control sample size will be mathematically split evenly to prevent artificial inflation of the sample size in the frequentist network model. Missing data will be sought by contacting study authors where feasible. If data remain unobtainable, the study will still be included in the meta-analysis using available data, and the missingness will be acknowledged as a limitation.

Crucially, before initiating the study, we will review high-quality clinical practice guidelines for substance use disorders to establish a rigorous, consensus-driven classification of NPI modalities. For independent trials operating outside the primary network due to unique counseling protocols or a focus on the sparse protracted withdrawal phase, descriptive data regarding compliance and therapeutic efficacy will be systematically extracted for narrative synthesis rather than quantitative pooling.

### Risk of bias assessment

Pairs of reviewers will independently assess the risk of bias for each eligible study using the revised Cochrane Risk of Bias tool for randomized trials (RoB 2.0) (15). Bias will be evaluated across five standard domains: (1) bias arising from the randomization process; (2) bias due to deviations from intended interventions; (3) bias due to missing outcome data; (4) bias in measurement of the outcome; and (5) bias in selection of the reported result. Each domain will be rated as “Low risk of bias”, “Some concerns”, or “High risk of bias”, culminating in an overall risk-of-bias judgment for each specific outcome based on predefined decision rules. Given the intrinsic nature of NPIs, blinding of participants and therapists is generally unfeasible. To prevent artificial inflation of risk in Domain 2, studies will be graded as “Some concerns” rather than “High risk” provided that strict adherence to the protocol environment was preserved without post-randomization deviations.

Crucially, to maintain a conservative and rigorous evidence synthesis, an overall judgment of “High risk of bias” will be automatically assigned to an outcome if either Domain 1 (specifically regarding allocation concealment) or Domain 3 (missing outcome data) is rated as high risk, recognizing that these domains represent critical vulnerabilities when double-blinding is absent.

### Data synthesis and statistical analysis

It is important to note that while study selection is complete, no results synthesis or evidence grading has commenced at the time of manuscript submission. All analytical procedures will be performed strictly according to the pre-specified plan described below. When mean change and standard deviations (SDs) are reported, we will utilize them directly. For studies providing pre- and post-intervention measures, we will apply the Cochrane Handbook methods to compute mean change and the corresponding SDs of change. If SDs are absent, they will be derived from standard errors, *p*-values, confidence intervals, or graphical data (16). For independent studies that do not meet the criteria for quantitative network analysis, such as behavioral trials lacking standardized numerical outcomes, we will conduct a qualitative, narrative synthesis of their clinical findings.

Dichotomous outcomes (e.g., relapse rates) will be pooled as risk ratios (RRs) with 95% confidence intervals (CIs). In contrast, given the expected rarity and heterogeneity of adverse events (AEs) in NPIs, AE data will not be meta-analysed. Instead, AEs will be summarised narratively in a structured table (any AE, serious AEs, and common specific AEs per study), with descriptive comparisons only. Continuous outcomes (anxiety, depression, and PSQI scores) will be expressed as Mean Differences (MDs) with 95% CIs. If these psychological or physiological outcomes are conceptually identical but measured using different clinical scales, Standardized Mean Differences (SMDs) with 95% CIs will be utilized. In addition, absolute treatment effects will be calculated for core outcomes. Pairwise meta-analyses using the random-effects model will be performed to obtain pooled direct effect estimates for each comparison featuring at least two independent trials. If 10 or more studies are available for a specific outcome, funnel plots and Egger’s tests will be implemented to investigate potential publication bias. The assessment of heterogeneity will be guided by the I^2^ statistic per the Cochrane Handbook, with statistical significance formally evaluated via *p*-values from the Chi^2^ test.

The entire analysis of the frequentist network meta-analysis will be executed using R (version 4.4.3). The threshold for statistical significance will be set at a two-tailed *p* ≤ 0.05. The primary network estimator will be based on random-effects multivariate meta-regression implemented through the Restricted Maximum Likelihood (REML) method to robustly account for between-study variability and evaluate heterogeneity across NPI nodes. We will utilize node-splitting approaches to estimate indirect effect sizes with their corresponding 95% CIs, allowing for the rigorous evaluation of local inconsistency between direct and indirect evidence. The transitivity assumption underpinning the indirect comparisons will be systematically assessed by evaluating the distribution of potential clinical effect modifiers (e.g., baseline dependence severity) across treatment comparisons using NMAstudio (https://www.nmastudioapp.com/).

To verify the robustness of our primary network estimates, sensitivity analyses will be performed by systematically excluding: (1) trials with high risk; (2) small-sample studies with extreme statistical imprecision (e.g., highly inflated standard errors) that threaten the stability of the model; and (3) trials with an overall participant attrition/dropout rate exceeding 20%.

To explore potential sources of heterogeneity and evaluate which clinical subpopulations are more sensitive to specific NPIs, network meta-regressions will be primarily executed, supplemented by subgroup analyses where data density permits, by integrating pre-specified effect modifiers. Subgroup variables were pre-specified based on prior RCT evidence and pilot search findings. Based on the robust baseline characteristics of addiction medicine literature, we will systematically examine primary potential effect modifiers as followed: (1) Background Opioid Agonist Treatment (OAT) Modality: To explore whether NPI efficacy is modulated by underlying pharmacological stabilizing mechanisms, studies will be stratified based on the concurrent maintenance regimen: Full Agonist Maintenance (e.g., methadone standalone), Partial Agonist/Antagonist Care (e.g., buprenorphine or naltrexone), or Combination/Flexible Pharmacotherapy protocols. (2) Summary Methodological Quality (Risk of Bias Status): To prevent artificial inflation of treatment effects driven by methodological vulnerabilities, a conventional, non-extreme classification rule will be applied to evaluate the modifying effect of study quality. (3) Intervention and Follow-up time: To evaluate time-dependent therapeutic decay, we will analyze the duration of NPI protocols (e.g., short-term vs. extended interventions) and differentiate follow-up periods into short-term and long-term assessment windows. Additionally, as a secondary clinical exploration to evaluate population-level vulnerability, covariate-outcome links for psychiatric comorbidity (will be explored via meta-regression when feasible), treating the continuous baseline percentage of participants with diagnosed severe psychiatric co-occurring disorders as a continuous covariate. These exploratory analyses will mathematically pinpoint the boundaries of NPI therapeutic responsiveness, directly informing personalized clinical placement for substance use disorders.

All statistical analyses will be performed under the supervision of a qualified biostatistician with expertise in meta-analysis methodology.

### Categorisation of interventions

To provide clinically actionable guidance, we will rank the evaluated NPIs from most to least effective using a minimally contextualized approach, integrating both the frequentist network effect estimates and the GRADE certainty of evidence (17). For the efficacy, we will classify interventions into three groups:

1. Baseline/Ineffective: The standard medication-based control group (e.g., methadone or buprenorphine standalone care) or NPI nodes demonstrated to be statistically equivalent to this control, designated as the “least effective” for those specific endpoints;
2. Moderately Effective: NPIs that yield statistically superior therapeutic gains compared to the medication-based control, yet do not achieve the top-tier performance threshold defined in (3);
3. Highly Effective: Premium NPI protocols that demonstrate statistically superior efficacy over at least one intervention in (2), representing the optimal clinical options within the network.

We will categorize the clinical utility of NPIs based on a critical matrix crossing their statistical effect sizes against whether they are supported by high/moderate versus low/very low GRADE certainty of evidence relative to the baseline reference node.

### Certainty of the evidence

The certainty of evidence for all direct, indirect, and network estimates will be evaluated using the GRADE framework, across five standard downgrading domains: risk of bias, inconsistency, indirectness, imprecision, and publication bias (18). For indirect evidence, the initial certainty rating will be determined by the lowest GRADE score among the direct comparisons contributing the greatest weight via the network’s contribution matrix. This baseline will be further downgraded if significant clinical intransitivity is identified via NMAstudio by assessing the distribution of core effect modifiers (such as background OAT modalities or baseline dependence severity) across trials.

The final network certainty rating will be based on the higher of the contributing direct and indirect estimates, with mandatory adjustments for imprecision and network inconsistency: (1) Imprecision Evaluation: Continuous endpoints (anxiety, depression, and PSQI sleep quality scores) will be downgraded for imprecision if their 95% CIs cross the minimally clinically important difference (MID) threshold. Given the variety of potential scales, we will define the MID as a standardized effect size of 0.5 × baseline pooled SD (moderate effect) for all continuous measures. This approach ensures a consistent and objective rule for downgrading. We will clearly document the actual MID values used for each scale in the final Summary of Findings table. For trials that do not report baseline SD, we will use the median SD from other included studies for that outcome, or if unavailable, we will not downgrade for imprecision and will discuss this limitation. The dichotomous endpoints (abstinence, relapse rates and treatment retention) will be downgraded if their 95% CIs cross the null effect (RR = 1.0), unless the sample size exceeds a strict optimal information size (N≧300). To avoid penalty duplication, estimates will not be downgraded twice if network inconsistency has already accounted for the statistical variance. (2) Small-Sample Effects: For critical endpoints informed by only a single isolated trial reporting a large, statistically significant effect, the evidence will be automatically flagged and downgraded due to potential small-sample artifacts.

In cases of unresolvable network inconsistency, the final clinical recommendation will prioritize the highest-quality evidence source between the direct and indirect estimates.

## Results

This study is designed as a network meta-analysis to synthesize and compare the available evidence on NPIs for OUD. The comprehensive literature search, data screening and extraction are currently underway, conducted strictly in accordance with the pre-specified analytical plan. All analytical procedures are scheduled for completion by December 2026, with subsequent manuscript preparation and submission for publication to follow immediately thereafter. As of the time of this protocol submission, data screening has been initiated, but no qualitative or quantitative synthesis, including network meta-analysis and GRADE certainty assessments, has commenced, thereby preserving the prospective nature of our analytical plan. The comprehensive findings will be fully reported once the statistical analyses are concluded and the final manuscript is accepted for publication. Figure 1 shows the research screening workflow diagram.

**Figure 1:**
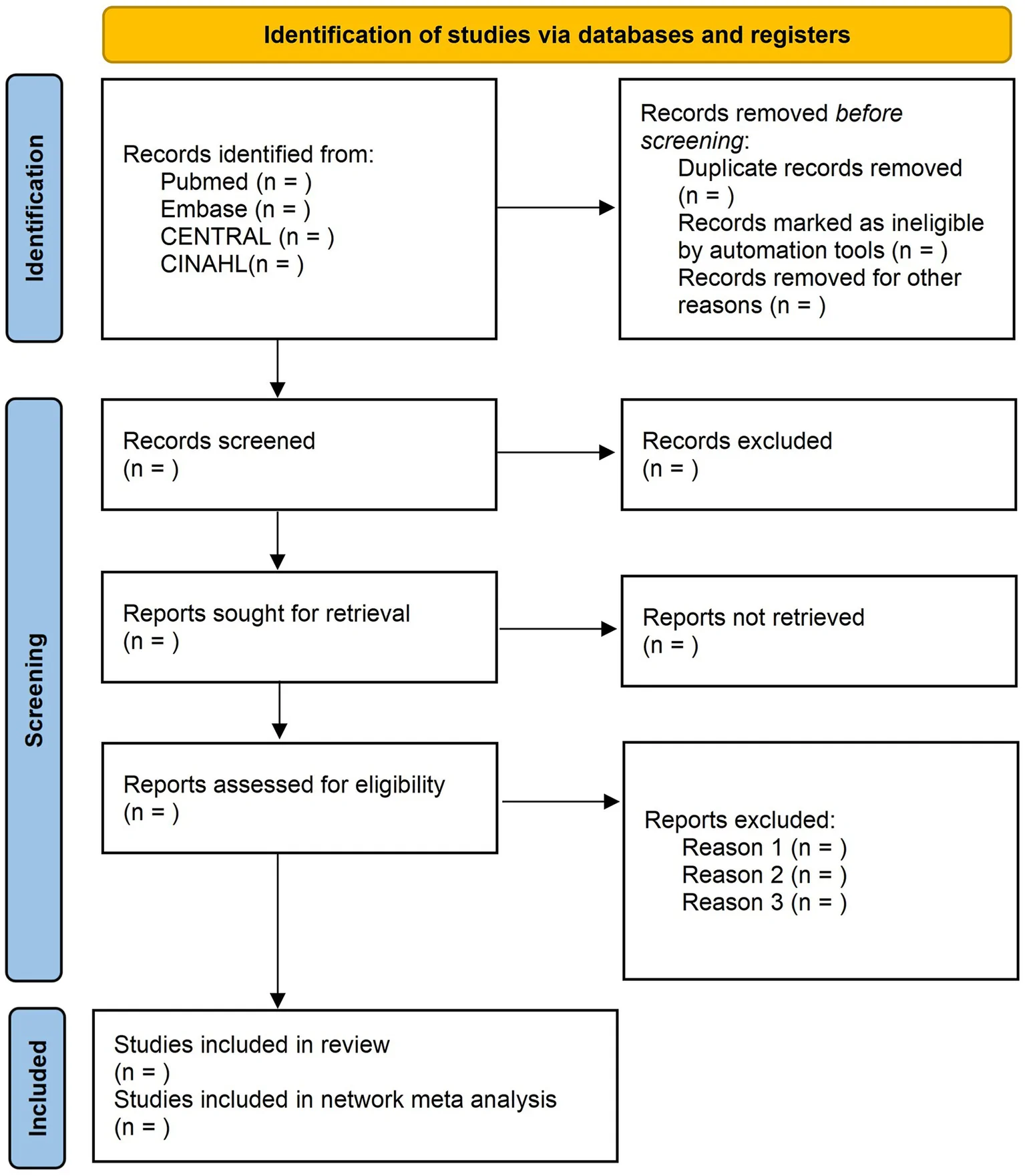
PRISMA diagram. This is the flowchart of the literature selection process for this study.

## Discussion

While methadone or buprenorphine maintenance therapies remain the undeniable cornerstone of OUD management, achieving long-term treatment retention and managing protracted withdrawal symptoms present substantial clinical hurdles. Even under optimal pharmacological stabilization, individuals with OUD frequently experience persistent psychiatric and physiological distress, most notably sleep disturbances, anxiety, or depression. These secondary symptoms heavily compromise patient adherence, driving high attrition and elevating the risk of serious relapse. NPIs, spanning manual-guided behavioral therapies, digital health applications, and neuromodulation techniques, offer promising, highly scalable adjunct options to address these residual domains. However, clinicians and policymakers currently lack a robust, evidence-based comparative hierarchy to determine which specific NPI protocol optimizes compliance and minimizes relapse for distinct OUD subpopulations, leading to fragmentation in clinical implementation.

Previous traditional meta-analyses on this topic are largely constrained by standard pairwise comparisons, which can only evaluate interventions that have been directly tested against one another in head-to-head trials. Consequently, the comparative efficacy of the rapidly expanding landscape of diverse NPI nodes remains fragmented and computationally isolated. This protocol expands upon prior research by utilizing a rigorous frequentist network framework to mathematically bridge direct and indirect evidence, thereby establishing a comprehensive, unified therapeutic hierarchy. Rather than generating a simple, linear ranking that overlooks clinical heterogeneity, this network meta-analysis is explicitly designed to handle the complex, multi-arm realities of addiction medicine trials, offering a global synthesis of available data that pairwise approaches cannot achieve. The pre‑specified subgroup and meta‑regression analyses transform the heterogeneity inherent in addiction medicine trials into clinically interpretable effect modifiers. This analytical strategy, informed by recent machine learning insights (19), aligns with the Rome Consensus’s emphasis on good design (20).

We anticipate several inherent methodological limitations within the included literature, most notably the structural unfeasibility of double-blinding in NPI trials; patients and therapists are naturally aware of behavioral or physical treatment assignments. To counter this structural vulnerability and prevent the artificial inflation of evidence certainty, our protocol implements a conservative Cochrane RoB 2.0 decision rule. Unlike conventional reviews that may overlook unblinded designs, an overall judgment of “High Risk of Bias” will be automatically assigned to any outcome if critical, blinding-resilient pillars, specifically allocation concealment (Domain 1) or missing outcome data (Domain 3), fail to meet rigorous standards.

By deciphering the modifier effects of background pharmacotherapies, summary methodological quality, baseline psychiatric comorbidities, and distinct intervention timepoints, the anticipated findings of this study will transition from abstract mathematical rankings into concrete, tier-structured clinical guidance. Through the application of a minimally contextualized framework, interventions will be structured into distinct clinical tiers that reflect both statistical effect sizes and evidence certainty relative to standard OAT controls. Ultimately, this study aims to bridge the current translational gap in addiction medicine by delivering a refined, data-driven selection matrix. This matrix will offer practical guidance for clinicians to select appropriate NPI protocols based on specific OAT regimen and the desired follow-up timeline, while providing a rigorous evidentiary foundation to inform the update of future clinical practice guidelines for substance use disorders. Emerging methodological frameworks, including biomimetic neurostimulation approaches and network-informed multi-trait genomic models, may inform future research by offering novel insights into neural modulation mechanisms and individualised treatment response (21, 22), although their direct clinical application to our review question remains exploratory.
