# Supplementary material for: Non-pharmacological interventions for opioid use disorder: protocol for a systematic review and network meta-analysis
Source: Front Public Health. 2026 Sep 15;14:1901002. doi: 10.3389/fpubh.2026.1901002 (PMC13619428; doi:10.3389/fpubh.2026.1901002)
Supplement: Supplementary file 1 [file Table_1.pdf]

Medline(PubMed)

	#	Searches	Results
**A. Opioid use disorder**	1	(Opioid-Related Disorder/[MeSH Terms]) OR (Opioid-Related Disorder[Title/Abstract] OR Opioid Dependence[Title/Abstract] OR Opioid Addiction[Title/Abstract] OR Opioid Abuse[Title/Abstract] OR Opioid Misuse[Title/Abstract] OR Opioid Use Disorder[Title/Abstract] OR Prescription Opioid Abuse[Title/Abstract] OR Prescription Opioid Misuse[Title/Abstract])	43886
	2	(Heroin Dependence[MeSH Terms]) OR (Heroin Dependence[Title/Abstract] OR Heroin Addiction[Title/Abstract] OR Heroin Smoking[Title/Abstract] OR Heroin Abuse[Title/Abstract])	10288
	3	(Morphine Dependence[Title/Abstract] OR Morphine Addiction[Title/Abstract] OR Morphine Abuse[Title/Abstract])	1264
	4	(Opium Dependence[MeSH Terms]) OR (Opium Dependence[Title/Abstract] OR Opium Addiction[Title/Abstract] OR Opium Smoking[Title/Abstract] OR Opium Abuse[Title/Abstract] OR Opium Use[Title/Abstract])	650
	5	((heroin*[Title/Abstract] OR analgesic*[Title/Abstract] OR opiate*[Title/Abstract] OR (opioid*[Title/Abstract] OR morphin*[Title/Abstract] OR methadone[Title/Abstract] OR oxycodone[Title/Abstract] OR oxycontin[Title/Abstract] OR narcot*[Title/Abstract] OR hydrocodone[Title/Abstract] OR hydromorphone[Title/Abstract] OR codeine[Title/Abstract] OR fentanyl[Title/Abstract] OR meperidine[Title/Abstract] OR oxymorphone[Title/Abstract] OR propoxyphene[Title/Abstract] OR tramadol[Title/Abstract] OR buprenorphine[Title/Abstract]) AND (abstin*[Title/Abstract] OR abus*[Title/Abstract] OR addict*[Title/Abstract] OR depend*[Title/Abstract] OR maintenance[Title/Abstract] OR detoxif*[Title/Abstract] OR overdos*[Title/Abstract] OR intoxicat*[Title/Abstract] OR withdraw*[Title/Abstract] OR relaps*[Title/Abstract])	92404
	6	#1 OR #2 OR #3 OR #4 OR #5	106874
**B1.Non-pharmacological treatment （Psychological interventions）**	7	(cognitive behavioral therapy[MeSH Terms]) OR (Cognitive Behavioral Therap*[Title/Abstract] OR Cognitive?Therap*[Title/Abstract] OR Cognitive Psychotherap*[Title/Abstract] OR Cognitive Behaviour Therap*[Title/Abstract])	50819
	8	(Behavior Therapy[MeSH Terms]) OR (Behavior Therapie*[Title/Abstract] OR Behavior Change Technique*[Title/Abstract] OR Behavior Treatment[Title/Abstract] OR Conditioning Therap*[Title/Abstract] OR Behavior Modification*[Title/Abstract] OR Behavior-Change Intervention*[Title/Abstract] OR Behavioral Counseling[Title/Abstract] OR Behavioral Support[Title/Abstract] OR Behavioral Technique*[Title/Abstract])	103638
	9	(Mindfulness-Based Cognitive Therapy[MeSH Terms]) OR (Mindfulness-Based Cognitive Therapy[Title/Abstract])	4006
	10	(Psychotherapy[MeSH Terms]) OR (Psychotherap*[Title/Abstract])	251531
	11	(Mindfulness[MeSH Terms]) OR (Mindfulness Meditation*[Title/Abstract])	9103
	12	Counseling[MeSH Terms]	50921
	13	(Motivational Interviewing[MeSH Terms]) OR (Motivational Interviewing[Title/Abstract])	6692
	14	(Social support[MeSH Terms]) OR (Social Support*[Title/Abstract] OR Social Care[Title/Abstract] OR Online Social Support*[Title/Abstract] OR Perceived Social Support*[Title/Abstract])	130161
	15	(Spiritual practice*[Title/Abstract] OR Psychosocial Therapy[Title/Abstract] OR Non-pharmacological[Title/Abstract] OR Community Reinforcement[Title/Abstract] OR	1390779

		Contingency Management[Title/Abstract] OR Psychosocial Support[Title/Abstract] OR Psychosocial intervention*[Title/Abstract] OR Positive reinforcement strategie*[Title/Abstract] OR Computer delivery psychosocial intervention*[Title/Abstract] OR Extended counseling[Title/Abstract] OR Psychotherap*[Title/Abstract] OR psychosocial*[Title/Abstract] OR behaviour?therapy[Title/Abstract] OR reinforcement[Title/Abstract] OR motivation*[Title/Abstract] OR contingent*[Title/Abstract] OR biofeedback[Title/Abstract] OR education*[Title/Abstract] OR brief intervention[Title/Abstract] OR early intervention[Title/Abstract] OR minimal intervention[Title/Abstract] OR counsel*[Title/Abstract] OR cognitive therapy[Title/Abstract] OR family therapy[Title/Abstract] OR stress management training[Title/Abstract] OR supportive expressive therapy[Title/Abstract] OR neurobehavioral*[Title/Abstract] OR coping skill*[Title/Abstract] OR self-control training[Title/Abstract] OR Digital intervention*[Title/Abstract])	
	16	#7 OR #8 OR #9 OR #10 OR #11 OR #12 OR #13 OR #14 OR #15	1651817
**B2.Non-pharmacological treatment （Alternative therapies）**	17	(Complementary Therapies[MeSH Terms]) OR (Complementary Medicine[Title/Abstract] OR Alternative Medicine[Title/Abstract] OR Complementary Therap*[Title/Abstract] OR Alternative Therap*[Title/Abstract])	287124
	18	(Acupuncture[MeSH Terms]) OR (Acupuncture Treatment*[Title/Abstract] OR Pharmacopuncture[Title/Abstract] OR Pharmacoacupuncture Treatment[Title/Abstract] OR Acupotom*[Title/Abstract] OR Auricular Acupuncture*[Title/Abstract] OR Acupuncture Point*[Title/Abstract] OR Acupoint*[Title/Abstract] OR Acupuncture Anesthesia[Title/Abstract])	35943
	19	Electroacupuncture[MeSH Terms]	5587
	20	(Moxibustion[MeSH Terms]) OR (Moxibustion[Title/Abstract]) OR (Moxabustion[Title/Abstract])	4426
	21	(Massage[MeSH Terms]) OR (Massage?Therap*[Title/Abstract] OR Zone Therap*[Title/Abstract])	8264
	22	(Cupping Therapy[MeSH Terms]) OR (Cupping Therap*[Title/Abstract] OR Cupping Treatment*[Title/Abstract] OR baguan[Title/Abstract])	449
	23	(Qigong[MeSH Terms]) OR (Qigong[Title/Abstract] OR Qi Gong[Title/Abstract] OR Ch'i Kung[Title/Abstract])	1395
	24	(Tai Ji[MeSH Terms]) OR (Tai Chi[Title/Abstract] OR Tai Ji Quan[Title/Abstract] OR Taijiquan[Title/Abstract] OR T'ai Chi[Title/Abstract] OR Tai Chi Chuan[Title/Abstract])	3047
	25	(Deep Brain Stimulation[MeSH Terms]) OR (Deep Brain Stimulation*[Title/Abstract] OR Electrical Stimulation Brain[Title/Abstract])	19215
	26	Transcranial Magnetic Stimulation[MeSH Terms] OR Transcranial Magnetic Stimulation*[Title/Abstract]	24683
	27	(Transcranial Direct Current Stimulation[MeSH Terms]) OR (tDCS[Title/Abstract] OR Anodal Stimulation tDCS[Title/Abstract] OR Cathodal Stimulation tDCS[Title/Abstract] OR Transcranial Alternating Current Stimulation[Title/Abstract] OR Transcranial Random Noise Stimulation[Title/Abstract] OR Repetitive Transcranial Electrical Stimulation[Title/Abstract] OR Transcranial Electrical Stimulation[Title/Abstract])	10709
	28	(Meditation[MeSH Terms]) OR (Transcendental Meditation[Title/Abstract])	4442
	29	Baduanjin[Title/Abstract] OR tuina[Title/Abstract] OR turina[Title/Abstract]	807
	30	#17 OR #18 OR #19 OR #20 OR #21 OR #22 OR #23 OR #24 OR #25 OR #26 OR #27 OR #28 OR #29	343667
**B3.Non-pharmacological treatment**	31	(Exercise[MeSH Terms]) OR (Physical Exercise*[Title/Abstract] OR Aerobic Exercise*[Title/Abstract] OR Isometric Exercise*[Title/Abstract] OR Acute	398665

（**Physical therapies**）		Exercise*[Title/Abstract] OR Exercise Training[Title/Abstract] OR Physical Activity[Title/Abstract])	
	32	(Sport[MeSH Terms]) OR Athletic*[Title/Abstract])	246537
	33	#31 OR #32	515392
**B4.Non-pharmacological treatment （Lifestyle adjustments）**	34	(Diet Therapy[MeSH Terms]) OR (Diet Therap*[Title/Abstract] OR Dietary Modification*[Title/Abstract] OR Diet Therapy Restrictive[Title/Abstract] OR Dietary Restriction[Title/Abstract])	78386
	35	Sleep intervention*[Title/Abstract]	980
	36	#34 OR #35	76364
**B1+B2+B3+B4**	37	#16 OR #30 OR #33 OR #36	2429323
**A+B**	38	#6 AND #37	15159
**C. RCT filter**	39	(Randomized Controlled Trial[MeSH Terms] OR Clinical Trial[MeSH Terms] OR Equivalence Trial[MeSH Terms] OR Pragmatic Clinical Trial[MeSH Terms] OR RCT[Title/Abstract] OR Random*[Title/Abstract] OR Placebo[Title/Abstract])	1968670
**RCT-Results (A+B+C)**	42	#38 AND #39	2332

Cochrane Central Register of Controlled Trials

	No.	Query	Results
**B. Opioid use disorder**	1	[mh "Opioid-Related Disorder"]	1093
	2	("Opioid-Related Disorder" OR "Opioid Dependence" OR "Opioid Addiction" OR "Opioid Abuse" OR "Opioid Misuse" OR "Opioid Use Disorder" OR "Prescription Opioid Abuse" OR "Prescription Opioid Misuse"):ti,ab,kw	2737
	3	[mh "Heroin Dependencer"]	940
	4	("Heroin Dependence" OR "Heroin Addiction" OR "Heroin Smoking" OR "Heroin Abuse"):ti,ab,kw	985
	5	("Morphine Dependence" OR "Morphine Addiction" OR "Morphine Abuse"):ti,ab,kw	442
	6	[mh "Opium Dependence"]	15
	7	("Opium Dependence" OR "Opium Addiction" OR "Opium Smoking" OR "Opium Abuse" OR "Opium Use"):ti,ab,kw	144
	8	(heroin* OR analgesic* OR opiate* OR opioid* OR morphin* OR "methadone" OR "oxycodone" OR "oxycontin" OR narcot* OR "hydrocodone" OR "hydromorphone" OR "codeine" OR "fentanyl" OR "meperidine" OR "oxymorphone" OR "propoxyphene" OR "tramadol" OR "buprenorphine"):ti,ab,kw AND (abstin* OR abus* OR addict* OR depend* OR maintenance OR detoxif* OR overdos* OR intoxicat* OR withdraw* OR relaps*):ti,ab,kw	19378
	9	#1 OR #2 OR #3 OR #4 OR #5 OR #6 OR #7 OR #8	19878
**B1.Non-pharmacological treatment （Psychological interventions）**	10	[mh "cognitive behavioral therapy"]	22398
	11	("Cognitive Behavioral Therapy" OR "Cognitive Therapy" OR "Cognitive Psychotherapy" OR "Cognitive Behavior Therapy"):ti,ab,kw	26476
	12	[mh "Behavior Therapy"]	14820
	13	("Behavior Therapy" OR "Behavior Change Technique" OR "Behavior Treatment" OR "Conditioning Therapy" OR "Behavior Modification" OR "Behavior-Change Intervention" OR "Behavioral Counseling" OR "Behavioral Support" OR "Behavioral Technique"):ti,ab,kw	15864
	14	[mh "Mindfulness-Based Cognitive Therapy"]	1072

	15	(Mindfulness-Based Cognitive Therapy):ti,ab,kw	1001
	16	Psychotherapy	20707
	17	(Psychotherap*):ti,ab,kw	18439
	18	Mindfulness	11134
	19	("Mindfulness Meditation"):ti,ab,kw	1313
	20	Counseling	32073
	21	"Motivational Interviewing"	5586
	22	("Motivational Interviewing"):ti,ab,kw	5313
	23	"Social support"	11922
	24	("Social Support" OR "Social Care" OR "Online Social Support" OR "Perceived Social Support"):ti,ab,kw	12547
	25	("Spiritual practice" OR "Psychosocial Therapy" OR "Non-pharmacological" OR "Community Reinforcement" OR "Contingency Management" OR "Psychosocial Support" OR "Psychosocial intervention" OR "Positive reinforcement strategie" OR "Computer delivery psychosocial intervention" OR "Extended counseling" OR "Psychotherapy" OR "psychosocial" OR "behavior therapy" OR reinforcement OR motivation* OR contingent* OR biofeedback OR education* OR "brief intervention" OR "early intervention" OR "minimal intervention" OR counsel* OR "cognitive therapy" OR "family therapy" OR "stress management training" OR "supportive expressive therapy" OR neurobehavioral* OR "coping skill" OR "self-control training" OR "Digital intervention"):ti,ab,kw	217951
	26	#12 OR #13 OR #14 OR #15 OR #16 OR #17 OR #18 OR #19 OR #20 OR #21 OR #22 OR #23 OR #24 OR #25	242972
**B2.Non-pharmacological treatment （Alternative therapies）**	27	"Complementary Therapies"	2940
	28	("Complementary Medicine" OR "Alternative Medicine" OR "Complementary Therapy" OR "Alternative Therapy"):ti,ab,kw	4806
	29	Acupuncture OR Electroacupuncture OR Moxibustion OR Massage OR "Cupping Therapy" OR Qigong OR Tai Ji OR "Deep Brain Stimulation" OR "Transcranial Magnetic Stimulation" OR "Transcranial Direct Current Stimulation" OR Meditation	56224
	30	("Acupuncture Treatment" OR Pharmacupuncture OR "Pharmacupuncture Treatment" OR Acupotom* OR "Auricular Acupuncture" OR "Acupuncture Point" OR Acupoint* OR "Acupuncture Anesthesia"):ti,ab,kw	10247
	31	(Moxibustion OR Moxabustion):ti,ab,kw	2759
	32	(Massage?Therap* OR "Zone Therapy" OR "Cupping Therapy" OR "Cupping Treatment" OR baguan OR Qigong OR "Qi Gong" OR "Ch'i Kung" OR "Tai Chi" OR "Tai Ji Quan" OR Taijiquan OR "T'ai Chi" OR "Tai Chi Chuan"):ti,ab,kw	3279
	33	("Deep Brain Stimulation" OR "Electrical Stimulation Brain" OR "Transcranial Magnetic Stimulation" OR tDCS OR "Anodal Stimulation tDCS" OR "Cathodal Stimulation tDCS" OR "Transcranial Alternating Current Stimulation" OR "Transcranial Random Noise Stimulation" OR "Repetitive Transcranial Electrical Stimulation" OR "Transcranial Electrical Stimulation" OR "Transcendental Meditation"):ti,ab,kw	16398
	34	(Baduanjin OR tuina OR turina):ti,ab,kw	818
	35	#27 OR #28 OR #29 OR #30 OR #31 OR #32 OR #33 OR #34	65812
**B3.Non-pharmaco**	36	Exercise OR Sport	153110

**logical treatment （Physical therapies）**	37	("Physical Exercise" OR "Aerobic Exercise" OR "Isometric Exercise" OR "Acute Exercise" OR "Exercise Training" OR "Physical Activity" OR Athletic):ti,ab,kw	76159
	38	#36 OR #37	177277
**B4.Non-pharmacological treatment （Lifestyle adjustments）**	39	"Diet Therapy"	14360
	40	("Diet Therapy" OR "Dietary Modification" OR "Diet Therapy Restrictive" OR "Dietary Restriction"):ti,ab,kw	15070
	41	("Sleep intervention"):ti,ab,kw	519
	42	#39 OR #40 OR #41	15700
**B1+B2+B3+B4**	43	#26 OR #35 OR #38 OR #42	440653
**A+B**	44	#9 AND #43	4264
**C. filter**	45	Conference proceeding:pt or abstract:so	258601
**D. Results**	46	(#44 not #45) in Cochrane Reviews and Trials	3839

Embase.com

	No.	Query	Results
**A:Opioid use disorder**	#1	'opioid use disorder'/exp	52427
	#2	('opioid* addict*' OR 'opioid* crisis' OR 'opioid* dependen*' OR 'opioid* epidemic' OR 'opioid use disorder' OR 'heroinism'):ti,ab,kw	28416
	#3	'heroin dependence'/exp	10743
	#4	'morphine addiction'/exp	4019
	#5	('morphine addict*' OR 'morphine dependen*'):ti,ab,kw	2925
	#6	'opiate addiction'/exp	33980
	#7	('opiate crisis' OR 'opiate dependence' OR 'opiate epidemic' OR 'opiate addiction'):ti,ab,kw	3028
	#8	'opioid craving'/exp	18
	#9	'opioid craving':ti,ab,kw	281
	#10	'opioid-related disorder'/exp	49047
	#11	('opioid* associated disorder' OR 'opioid* induced disorder' OR 'opioid-related disorder*'):ti,ab,kw	1144
	#12	'opioid intoxication'/exp	81
	#13	('opioid* toxicity' OR 'opioid* intoxication'):ti,ab,kw	703
	#14	'opioid poisoning'/exp	32
	#15	('opioid* intoxication' OR 'opioid toxidrome' OR 'opioid* poisoning'):ti,ab,kw	569
	#16	'opioid withdrawal syndrome'/exp	149
	#17	('opioid* withdrawal' OR 'opioid withdrawal syndrome'):ti,ab,kw	3673
	#18	((analgesic* OR morfin* OR methadone OR oxycodone OR oxycontin OR narcot* OR hydrocodone OR hydromorphone OR codeine OR fentanyl OR meperidine OR oxymorphone OR propoxyphene OR tramadol OR buprenorphine) and (abstin* or abstinen* or abus* or addict* or depend* OR maintenance or detoxif* or desintoxi* or disintoxi* or disintossi* or overdos* or intoxicat* or withdraw* or relaps*)):ti,ab,kw	69391
	#19	#1 OR #2 OR #3 OR #4 OR #5 OR #6 OR #7 OR #8 OR #9 OR #10 OR #11 OR #12 OR #13 OR #14 OR #15 OR #16 OR #17 OR #18	115352
**B1.Non-pharmacological treatment （Psychological interventions）**	#20	'cognitive behavioral therapy'/exp	40415
	#21	("CBT" OR "cognitive behavior" OR "cognitive behaviour"):ti,ab,kw	34191
	#22	('Behavior Therapy' OR 'Mindfulness-Based Cognitive Therapy' OR 'Psychotherapy' OR 'Psychosocial intervention' OR 'Mindfulness' OR 'Mindfulness-based intervention' OR 'Counselling' OR 'Motivational Interviewing' OR 'Social support')/exp	683995
	#23	(('behavior' OR 'behaviour') AND ('therapy' OR 'treatment' OR 'psychotherapy')) :ti,ab,kw	260375
	#24	'mindfulness' AND ('CBT' OR 'cognitive therapy' OR 'cognitive behavior therapy' OR 'cognitive behaviour therapy' OR "intervention" OR "therapy"):ti,ab,kw	14899
	#25	('holistic psychotherapy' OR 'multiple psychotherapy' OR 'psychotherapeutic processes' OR 'psychotherapeutic training' OR 'socioenvironmental therapy'):ti,ab,kw	336
	#26	(('psycho-social' OR 'psychosocial') AND ('intervention' OR 'therapy' OR 'treatment')):ti,ab,kw	90667
	#27	('counselling' OR 'counseling' OR 'motivational interviewing' OR 'social support'):ti,ab,kw	282669

	#28	('spiritual practices' OR 'non-pharmacological' OR 'community reinforcement' OR 'contingency management' OR 'psychosocial support' OR 'positive reinforcement strategie*' OR 'brief intervention' OR 'family therapy' OR 'stress management training' OR 'supportive expressive therapy' OR 'neurobehavioral*' OR 'self-control training' OR 'digital intervention*'):ti,ab,kw	75153
	#29	#20 or #21 or #22 or #23 or #24 or #25 or #26 or #27 or #28	1106053
**B2.Non-pharmacological treatment (Alternative therapies）**	#30	('Complementary Therapies' OR 'Acupuncture' OR 'Electroacupuncture' OR 'Warm acupuncture' OR 'Auricular acupuncture' OR 'Tui Na' OR 'Moxibustion' OR 'Massage' OR 'Cupping Therapy' OR 'Qigong' OR 'Tai Ji' OR 'Brain depth stimulation' OR 'Transcranial Magnetic Stimulation' OR 'Ranscranial Direct Current Stimulation' OR 'Transcranial electrical stimulation' OR 'Meditation')/exp	272290
	#31	('alternative therap*' OR 'complementary medicine' OR 'complementary therap*' OR 'eclectic medicine' OR 'historical eclecticism' OR 'mental healing' OR 'mind body technique' OR 'mind body therap*' OR 'mind-body relations ' OR 'polarity therapy' OR 'radi?esthesia' OR 'therapeutic cults' OR 'thomsonian medicine' OR 'alternative medicine'):ti,ab,kw	61762
	#32	('needle therapy' OR 'fire acupuncture' OR 'fire needling' OR 'heat?acupuncture' OR 'therm?acupuncture' OR 'needle acupuncture' OR 'warm acupuncture'):ti,ab,kw	955
	#33	('earlobe acupuncture' OR 'auriculo?acupuncture' OR 'auriculotherapy' OR 'ear acupuncture'):ti,ab,kw	855
	#34	('massage' OR 'cupping'):ti,ab,kw	22569
	#35	('chi kung' OR 'chigung' OR 'qigong' OR 'Tai Ji' OR 'Tai Chi'):ti,ab,kw	5289
	#36	('brain excitation' OR 'brain stimul*' OR 'brain stimulation' OR 'brain stimulation' OR 'transcranial magnetic stimulation' OR 'tDCS' OR 'transcranial direct current stimulation'):ti,ab,kw	78828
	#37	('Baduanjin' OR 'tuina'):ti,ab,kw	1059
	#38	#30 OR #31 OR #32 OR #33 OR #34 OR #35 OR #36 OR #37	321441
**B3.Non-pharmacological treatment(Physical therapies)**	#39	'exercise' OR 'sport'/exp	946816
	#40	('exertion' OR 'fitness training' OR 'fitness workout' OR 'physical effort' OR 'physical exertion' OR 'physical workout' OR 'exercise' OR 'sport'):ti,ab,kw	579126
	#41	#39 OR #40	978529
**B4.Non-pharmacological treatment(Lifestyle adjustments)**	#42	'Diet Therapy'/exp	481634
	#43	('diet* intervention*' OR 'diet* treatment' OR 'diet* therapy' OR 'nutrition* therapy'):ti,ab,kw	46368
	#44	#42 OR #43	499395
**B1+B2+B3+B4**	#45	#29 OR #38 OR #41 OR #44	2716104
**A+B**	#46	#19 AND #45	15061
**C. RCT filter**	#47	'randomized controlled trial'/exp	997931
	#48	(Clinical Trial OR Equivalence Trial OR Pragmatic Clinical Trial OR RCT OR Random* OR Placebo):ti,ab,kw	2816833
	#49	#47 OR #48	2925945
**Results (A+B+C)**	#50	#46 AND #49	3321
	#51	#50 NOT ([preprint]/lim OR 'conference abstract'/it OR 'conference paper'/it OR 'conference review'/it)	2763

CINAHL

	No.	Query	Limiters/Expanders
**A:Opioid use disorder**	#1	SU ("Opioid Epidemic")	Search modes - Find all my search terms
	#2	SU "Heroin"	Search modes - Find all my search terms
	#3	SU "Morphine"	Search modes - Find all my search terms
	#4	SU ("Opiate Overdose" OR "Opioid Receptors" OR "Substance Abuse" OR "Substance Dependence")	Search modes - Find all my search terms
	#5	SU("Substance Withdrawal Syndrome" OR "Substance Withdrawal Controlled")	Search modes - Find all my search terms
	#6	TI ((heroin* OR analgesic* OR opiate* OR opioid* OR morphin* OR morfin* OR methadone OR oxycodone OR oxycontin OR narcot* OR hydrocodone OR hydromorphone OR codeine OR fentanyl OR meperidine OR oxymorphone OR propoxyphene OR tramadol OR buprenorphine) AND (abstin* or abus* or addict* or depend* OR maintenance or detoxif* or desintoxi* or disintoxi* or disintossi* or overdos* or intoxicat* or withdraw* or relaps*))	Search modes - Find all my search terms
	#7	TI("opioid poisoning" or "opioid use disorder" or "opioid craving" or "opioid-related disorder" or "opioid withdrawal syndrome")	Search modes - Find all my search terms
	#8	#1 OR #2 OR #3 OR #4 OR #5 OR #6 OR #7	Search modes - Find all my search terms
**B1.Non-pharmacological treatment(Psychological interventions)**	#9	SU("Cognitive Therapy" OR "Behavior Therapy")	Search modes - Find all my search terms
	#10	SU(Psychotherapy" OR "Psychosocial Intervention")	Search modes - Find all my search terms
	#11	SU("Mindfulness" OR "Mind Body Techniques" OR "Acceptance and Commitment Therapy")	Search modes - Find all my search terms
	#12	SU("Counseling")	Search modes - Find all my search terms
	#13	SU("Motivational Interviewing" OR "Craving")	Search modes - Find all my search terms
	#14	SU("Social Support " OR "Family Support" OR "Community Support" OR " Psychosocial Support")	Search modes - Find all my search terms
	#15	SU("Early Intervention" OR "Brief Psychotherapy" OR "Internet-Based Intervention")	Search modes - Find all my search terms
	#16	SU("Digital Health")	Search modes - Find all my search terms
	#17	TI("Spiritual practices" OR "Psychosocial Therapy" OR "Non-pharmacological" OR "Community Reinforcement" OR "Contingency Management" OR "Positive reinforcement strategy" OR "family therapy" OR "stress management training" OR "supportive expressive therapy" OR "coping skill" OR "self-control training" OR "Digital intervention" OR "mindfulness-based intervention")	Search modes - Find all my search terms

**B2.Non-pharmacological treatment (Alternative therapies）**	#18	SU("Alternative Therapies" OR "Massage Therapy" OR "Cupping Therapy")	Search modes - Find all my search terms
	#19	SU("Acupuncture" OR "Dry Needling" OR "Electroacupuncture" OR "Acupressure" OR "Auriculotherapy" OR "Acupuncture Points")	Search modes - Find all my search terms
	#20	SU("Tui Na" OR "Moxibustion" OR "Massage" OR "Physical Therapists" OR "Meditation" OR "Mindfulness" OR MH "Yoga" OR "Qigong" OR "Tai Chi")	Search modes - Find all my search terms
	#21	SU("Deep Brain Stimulation" OR "Electric* Stimulation" OR "Transcranial Magnetic Stimulation" OR"Transcutaneous Electric Nerve Stimulation" OR "tDCS" OR "transcranial direct current stimulation")	Search modes - Find all my search terms
	#22	TI("Complementary Therapies" OR "Baduanjin")	Search modes - Find all my search terms
**B3.Non-pharmacological treatment(Physical therapies)**	#23	SU("Physical Therapy" OR "Exercise")	Search modes - Find all my search terms
**B4.Non-pharmacological treatment(Lifestyle adjustments)**	#24	SU("Diet Therapy")	Search modes - Find all my search terms
**B1+B2+B3+B4**	#25	#9 OR #10 OR #11 OR #12 OR #13 OR #14 OR #15 OR #16 OR #17 OR #18 OR #19 OR #20 OR #21 OR #22 OR #23 OR #24	Search modes - Find all my search terms
**C:RCT**	#26	SU("Clinical Trials+")	Search modes - Find all my search terms
	#27	SU("Random Assignment")	Search modes - Find all my search terms
	#28	SU("Placebos")	Search modes - Find all my search terms
	#29	TI("randomized controlled trial" or "controlled clinical trial" or "randomized" or "placebo" or "clinical trial" or "random*" or "trial")	Search modes - Find all my search terms
	#30	#30 OR #31 OR #32 OR #33	Search modes - Find all my search terms
**A+B+C**	#31	#8 AND #25 OR #30	Search modes - Find all my search terms
